# CD3xPDL1 bi-specific T cell engager (BiTE) simultaneously activates T cells and NKT cells, kills PDL1^+^ tumor cells, and extends the survival of tumor-bearing humanized mice

**DOI:** 10.18632/oncotarget.19865

**Published:** 2017-08-03

**Authors:** Lucas A. Horn, Nicholas G. Ciavattone, Ryan Atkinson, Netsanet Woldergerima, Julia Wolf, Virginia K. Clements, Pratima Sinha, Munanchu Poudel, Suzanne Ostrand-Rosenberg

**Affiliations:** ^1^ Department of Biological Sciences, University of Maryland Baltimore County, Baltimore, MD, USA; ^2^ Marlene and Stewart Greenebaum Comprehensive Cancer Center, University of Maryland, Baltimore, MD, USA

**Keywords:** tumor-induced immune suppression, checkpoint inhibitor blockade, T cell activation, solid tumors, cancer immunotherapy

## Abstract

Bi-specific T cell engagers (BiTEs) activate T cells through CD3 and target activated T cells to tumor-expressed antigens. BiTEs have shown therapeutic efficacy in patients with liquid tumors; however, they do not benefit all patients. Anti-tumor immunity is limited by Programmed Death 1 (PD1) pathway-mediated immune suppression, and patients who do not benefit from existing BiTES may be non-responders because their T cells are anergized via the PD1 pathway. We have designed a BiTE that activates and targets both T cells and NKT cells to PDL1^+^ cells. *In vitro* studies demonstrate that the CD3xPDL1 BiTE simultaneously binds to both CD3 and PDL1, and activates healthy donor CD4^+^ and CD8^+^ T cells and NKT cells that are specifically cytotoxic for PDL1^+^ tumor cells. Cancer patients’ PBMC are also activated and cytotoxic, despite the presence of myeloid-derived suppressor cells. The CD3xPDL1 BiTE significantly extends the survival time and maintains activated immune cell levels in humanized NSG mice reconstituted with human PBMC and carrying established human melanoma tumors. These studies suggest that the CD3xPDL1 BiTE may be efficacious for patients with PDL1^+^ solid tumors, in combination with other immunotherapies that do not specifically neutralize PD1 pathway-mediated immune suppression.

## INTRODUCTION

Bi-specific T cell engagers (BiTEs) are genetically engineered recombinant proteins that have been shown to simultaneously activate cytotoxic T cells through the T cell CD3 complex and target the activated T cells to tumor cells. They are single chain molecules that consist of the V_L_ and V_H_ chains of an anti-CD3 and an anti-tumor antigen antibody that are connected by short linker sequences [1]. BiTEs do not require pre-activation of T cells [2, 3], and since they activate through the CD3 complex, their function is independent of T cell receptor specificity, MHC restriction, and costimulatory signals [4–6]. BiTEs are relatively small in size (~55kD) so they effectively bridge T cells to target cells [6]. The short bridging distance factors into the potency of the BiTE [7] and facilitates the formation of an immunological synapse that favors T cell to target cell interaction [6]. BiTEs kill target cells through a granzyme and perforin-mediated process [8], and they convert T regulatory cells to cytotoxic T cells [9].

BiTEs have been designed to target a variety of tumor antigens such as CD19 [2, 10, 11], EphA2 receptor tyrosine kinase [12], EpCAM [13, 14], EGFR [15], melanoma-associated chondroitin sulfate proteoglycan [16], and CD33 [17], among others, and have shown therapeutic efficacy in mouse tumor models [3, 4, 13, 14]. Blincyto (blinatumomab), which consists of anti-CD3 and anti-CD19 V_L_ and V_H_ regions [2], is the first BiTE to be used clinically and was approved by the FDA in 2014 for the treatment of acute lymphoblastic leukemia.

T cell activation to tumor antigens is a major factor in mobilizing an individual's immune system to eliminate cancer cells. However, once activated, T cells must remain activated to efficiently eliminate tumor cells. Abundant studies in both mouse models and cancer patients have demonstrated that tumor cells contribute to a profound state of immune suppression in many, if not most, individuals with cancer [18]. Tumor-induced immune suppression is mediated by cell populations including myeloid-derived suppressor cells (MDSC) (reviewed in [19]) and T regulatory cells [20], as well as by checkpoint inhibitors which cause T cell anergy and apoptosis [21]. Clinical trials with antibodies to checkpoint inhibitors revealed that the programmed death 1 (PD1) pathway is a major contributor to immune suppression in melanoma, renal cell carcinoma, and non-small cell lung cancer patients since antibodies to either the receptor on T cells (PD1) or the ligand for the receptor (PDL1) significantly increased patient survival.

Although antibodies to PD1 and PDL1 have dramatic clinical effects in patients, their efficacy is limited to a subset of cancer patients, and only approximately 15-30% of patients benefit [22]. The efficacy of these antibodies depends on patients having naturally activated T cells that kill cancer cells since the PD1 pathway comes into play only after T cells are activated [21]. However, many cancer patients do not spontaneously develop tumor-reactive T cells. Some of the approximately 70%-80% of melanoma, renal cell carcinoma, and non-small cell lung cancer patients who do not benefit from checkpoint inhibitor therapy may be non-responders because they do not have naturally tumor-reactive T cells. PD1 pathway suppression is also implicated in reducing BiTE efficacy in that acute lymphoblastic leukemia patients with increased levels of PDL1 do not benefit from Blincyto therapy [23]. To overcome this critical limitation, it is essential to develop therapies that not only neutralize PD1-mediated immune suppression, but also activate T cells that kill cancer cells. Given the efficacy of BiTEs for activating T cells, we are developing a BiTE that simultaneously neutralizes PD1-mediated immune suppression, while simultaneously activating T cells and targeting them to tumor cells.

Our novel BiTE consists of the V_L_ and V_H_ chains of anti-CD3 monoclonal antibody (mAb) linked to the V_L_ and V_H_ chains of anti-PDL1 mAb. This BiTE has the potential to not only activate cytotoxic T cells, but to concomitantly bridge activated T cells to PDL1-expressing tumor cells and thereby eliminate tumor cells that drive PD1 immune suppression. Our *in vitro* studies demonstrate that the CD3xPDL1 BiTE efficiently and specifically activates not only CD4^+^ and CD8^+^ T cells, but also CD3^+^ NKT cells from healthy donors, and that PBMC from cancer patients are also activated. Activated PBMC produce IFNγ and are cytotoxic for multiple PDL1^+^ human tumor cells. *In vivo* studies demonstrate that the CD3xPDL1 BiTE significantly prolongs the mean survival time (MST) of NSG mice carrying established spontaneously metastatic human melanoma tumor and reconstituted with human PBMC.

## RESULTS

### Generation of the CD3xPDL1 BiTE

**Figure 1:**
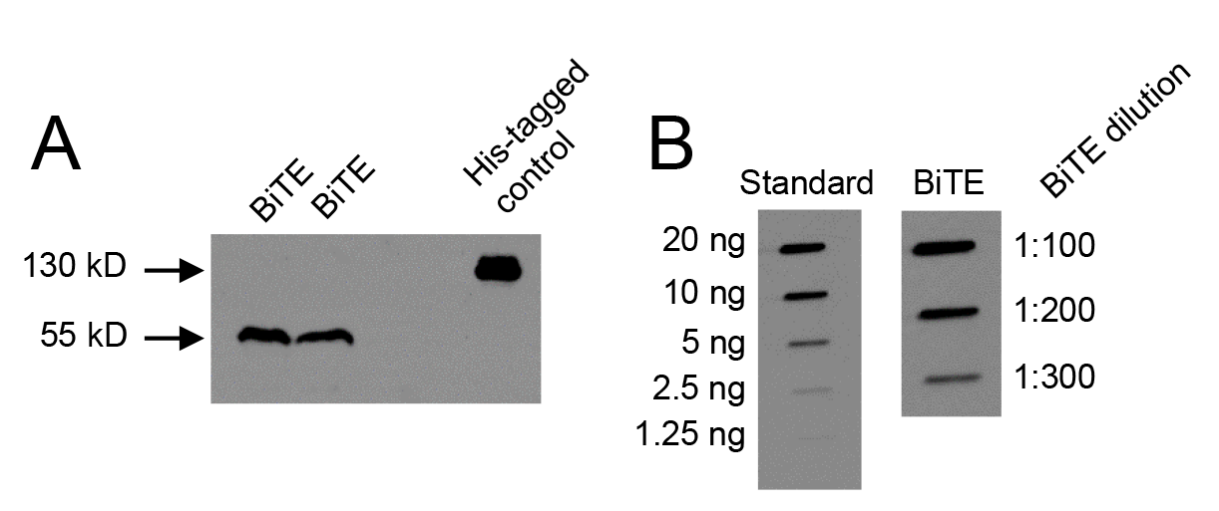
CHO cells transfected with the CD3xPDL1 BiTE produce a 55 kD protein **A**. Supernatants from BiTE-transfected CHO cells (CHO/BiTE) cultured in serum-free HL1 medium were concentrated using 10kD spin columns, electrophoresed by SDS-PAGE, and western blotted using an anti-His mAb. **B**. Supernatants of CHO/BiTE transfectants were slot-blotted, probed with anti-his mAb, and BiTE content quantified by comparing to a standard his-tagged protein (CD80-Fc) using ImageJ software.

The CD3xPDL1 BiTE was generated as a single chain Fv recombinant protein consisting of the V_L_ and V_H_ gene segments of the humanized OKT3 anti-CD3 monoclonal antibody [24, 25] and the V_H_ and V_L_ gene segments of the human 4A12 anti-PDL1 monoclonal antibody [26] cloned into the pINFUSE vector (Supplementary Figure 1A-D). The CD3xPDL1/pINFUSE construct was transfected into CHO cells, and stable BiTE-producing transfectants were obtained by selection on zeocin. Stable transfectants were grown in serum-free medium and produced a ~53KDa recombinant protein as assessed by western blotting using an anti-his mAb (Figure 1A; Supplementary Figure 1E). BiTE/CHO cells produced approximately 5-7.5 μg/10^7^ cells/10mL/3 days as assessed by slot blotting and ImageJ software analysis (Figure 1B). Biacore analysis demonstrated that the BiTE bound PDL1 with a KD of 1.28×10^-11^ and CD3 with a KD of 2.4×10^-10^ (Supplementary Figure 1F).

### The CD3xPDL1 BiTE binds specifically to PDL1^+^ tumor cells and CD3^+^ PBMC

**Figure 2:**
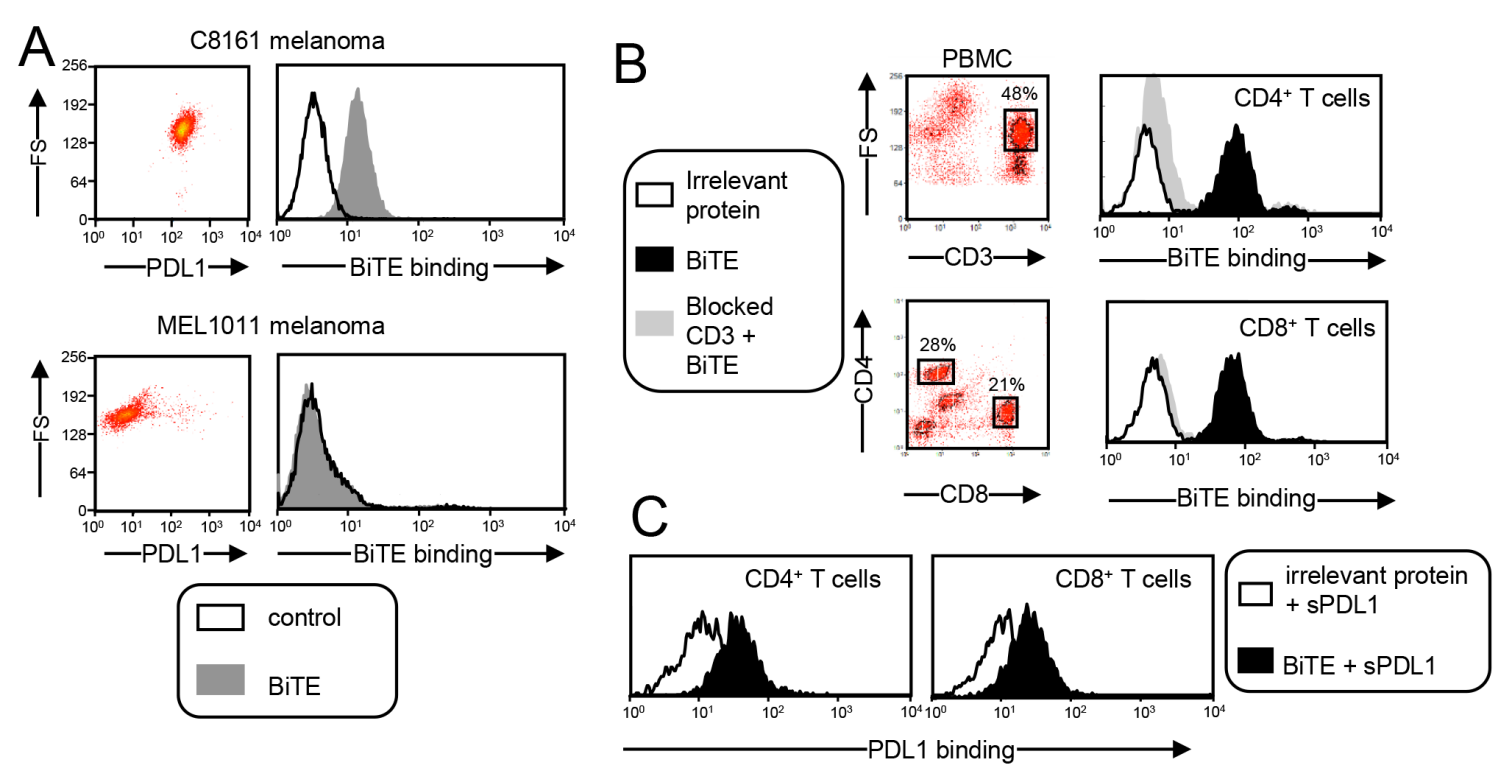
CD3xPDL1 BiTE simultaneously and specifically binds to PDL1**^+^** human tumor cells and CD3+ human T cells **A**. BiTE binds to tumor cell-expressed PDL1. PDL1^+^ C8161 melanoma cells and PDL1^-^ MEL1011 cells were stained with fluorescently-tagged antibodies to PDL1 to determine their level of PDL1 expression (left panels). C8161 and MEL1011 cells were incubated with the CD3xPDL1 BiTE or an irrelevant recombinant protein (TROY-Fc) and then stained with a fluorescently-tagged anti-his mAb to detect BiTE binding (right panels). **B**. BiTE binds to T cell-expressed CD3. Peripheral blood mononuclear cells (PBMC) from healthy human donors were stained with fluorescently-tagged antibodies to CD4 and CD8 followed by addition of BiTE or irrelevant recombinant protein, and then fluorescently tagged antibody to his to detect BiTE binding. To ascertain that the BiTE specifically binds to CD3, aliquots of PBMC were first blocked with antibodies to CD3 to prevent BiTE binding. **C**. BiTE simultaneously binds CD3 and PDL1. PBMC were stained for CD4 and CD8 as in panel B, and then incubated with either BiTE or an irrelevant protein followed by soluble PDL1 (sPDL1). PDL1 binding was detected with a fluorescently-tagged antibody to PDL1. Data are representative of more than three independent experiments with at least 3 batches of BiTE.

To ascertain that the CD3xPDL1 BiTE binds to PDL1 on cells, C8161 human melanoma cells that constitutively express PDL1 and PDL1^-^ MEL1011 cells were incubated with BiTE or an irrelevant his-tagged recombinant protein (TROY-Fc) followed by fluorescently tagged anti-his antibodies (Figure 2A). The BiTE bound to the PDL1^+^ cells and did not bind to the PDL1^-^ cells. To ascertain binding to CD3 on cells, PBMC from healthy human donors were stained with fluorescently labeled antibodies to CD4 and CD8, followed by either BiTE or an irrelevant recombinant protein (TROY-Fc), and then fluorescently tagged anti-his antibodies (Figure 2B). To confirm that the binding was specific for CD3, aliquots of PBMC were first saturated with unlabeled antibodies to CD3 to prevent BiTE binding, followed by BiTE or TROY-Fc, and then fluorescently tagged anti-his antibodies. BiTE bound to CD3^+^ PBMC only if the CD3 was not saturated with unlabeled anti-CD3 antibodies. To assess if the BiTE could simultaneously bind both CD3 and PDL1, CD3^+^ PBMC were stained for CD4 and CD8, followed by BiTE or TROY-Fc and soluble PDL1 (sPDL1). PDL1 binding was quantified by subsequent staining with fluorescently tagged antibodies to PDL1 (Figure 2C). CD4^+^ and CD8^+^ T cells incubated with the BiTE, but not with the irrelevant protein bound PDL1. These results demonstrate that the CD3xPDL1 BiTE binds simultaneously to CD3 and PDL1 and does not bind to cells lacking CD3 and PDL1.

### The CD3xPDL1 BiTE activates T cells that are specifically cytotoxic for PDL1^+^ tumor cells

**Figure 3:**
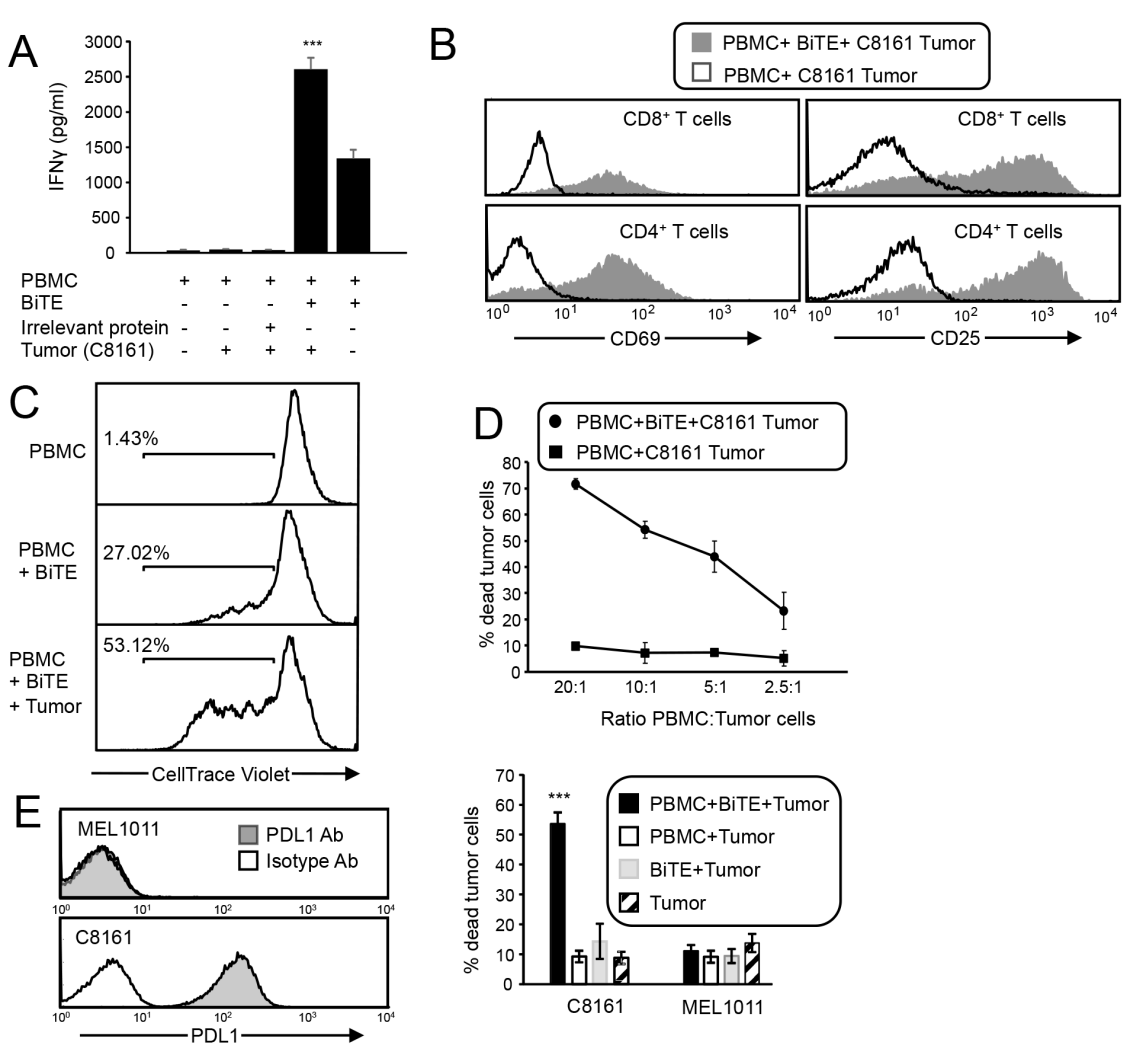
CD3xPDL1 BiTE activates T cells that are cytotoxic for PDL1 **^+^** tumor cells **A**. Healthy donor PBMC were co-cultured with C8161 tumor cells (ratio 20:1 PBMC:tumor cells) and with CD3xPDL1 BiTE (200 ng/mL) or an irrelevant protein for 48 hrs and the supernatants were analyzed for IFNγ by ELISA. Data are representative of one of two independent experiments. **B**. PBMC were incubated for 72 hrs with CellTrace Violet-labelled PDL1^+^ C8161 melanoma cells ± BiTE, and then labeled with fluorescently-coupled antibodies to CD4, CD8, and CD69 or CD25. Violet^+^ tumor cells were gated out and CD4^+^ and CD8^+^ T cells were gated and analyzed for the activation markers CD69 and CD25. Data are representative of 3 independent experiments. **C**. CellTrace Violet-stained CD3 purified PBMC were co-cultured for four days with or without BiTE or tumor, and the gated violet^+^ cells assessed by flow cytometry for proliferation. Data are representative of one of two independent experiments. **D**. C8161 cells were stained with CellTrace Violet and incubated at varying ratios with healthy donor PBMC ± BiTE. Following 72 hrs of incubation, cells were stained with the viability dye 7AAD. % dead tumor cells = [dead tumor cells (violet^+^7AAD^+^)/total tumor cells (violet^+^)] x 100%. Values are the average of triplicates per sample. **E**. C8161 and MEL1011 melanoma cells were stained with fluorescent antibodies to PDL1 (29E.2A3 mAb) or an isotype control mAb and analyzed by flow cytometry. PBMC from healthy donors were incubated ± CellTrace Violet-labeled C8161 or MEL1011 tumor cells ± BiTE and analyzed for % dead tumor cells. PBMC:tumor cell ratio is 20:1. CD8:tumor cell ratio is ~4:1. Values are the average + SE of 7 and 5 independent experiments for C8161 and MEL1011, respectively. For panels A and E, values with asterisks are significantly different from all other values.

To assess if the CD3xPDL1 BiTE activates T cells, PBMC from healthy donors were cultured with BiTE or an equal amount of irrelevant TROY-Fc recombinant protein in the presence or absence of PDL1^+^ human melanoma C8161 cells. T cell activation was measured 48 hours later by assessing production of interferon gamma (IFNγ) (Figure 3A). The BiTE induced high levels of IFNγ in the PBMC + BiTE + tumor cell samples. Significantly less IFNγ was produced in PBMC plus BiTE samples, and essentially no IFNγ was produced in the absence of BiTE, demonstrating that the BiTE activates PBMC from healthy donors. PBMC co-cultured with or without BiTE in the presence of tumor cells were also assessed for the activation markers CD69 and CD25. Both markers were upregulated on CD4^+^ and CD8^+^ T cells in the presence of BiTE and tumor, but were not upregulated in the absence of BiTE (Figure 3B). CD69 and CD25 were also upregulated by BiTE in the absence of PDL1^+^ tumor cells (Supplementary Figure 2). To determine if the BiTE drives PBMC proliferation, CellTrace Violet stained PBMC were co-cultured for four days with or without BiTE or tumor, and the resulting cells assessed by flow cytometry (Figure 3C). Approximately 50% of the PBMC proliferated in the presence of BiTE and tumor, whereas only 27% proliferated in the presence of BiTE, and 1.4% in the absence of BiTE and tumor.

To determine if the CD3xPDL1 BiTE kills PDL1^+^ tumor cells, PBMC from healthy donors were incubated at varying ratios with CellTrace Violet-labeled PDL1^+^ C8161 cells in the presence or absence of BiTE. After 48 hrs the cells were incubated with the viability dye 7AAD (Figure 3D). Approximately 70% of PDL1^+^ melanoma cells were killed in the presence of BiTE at the 20:1 ratio, and killing titered out to ~25% at the 2.5:1 ratio. Background levels of killing in the absence of BiTE or PBMC were <10%. To determine if the CD3xPDL1 BiTE was more effective than antibodies in activating tumoricidal PBMC, we compared the percent of dead C8161 cells following incubation with PBMC plus BiTE vs. PBMC plus anti-PDL1 mAb or PBMC plus anti-CD3 mAb (Supplementary Figure 3). The BiTE was significantly more effective than the anti-CD3 mAb and the PDL1 mAb has no killing above background.

To ascertain that the BiTE cytotoxic activity is specific for PDL1^+^ target cells, PDL1^-^ human MEL1011 cells were included in a cytotoxicity assay along with C8161 cells (Figure 3E). Less than 11% of MEL1011 cells were killed vs. >50% of PDL1^+^ C8161 cells. These results demonstrate that the BiTE in conjunction with PBMC efficiently kills PDL1^+^ tumor cells and does not have significant off-target effects on PDL1^-^ tumor cells.

### The BiTE activates PBMC that are cytotoxic for multiple PDL1^+^ human tumor cells

**Figure 4:**
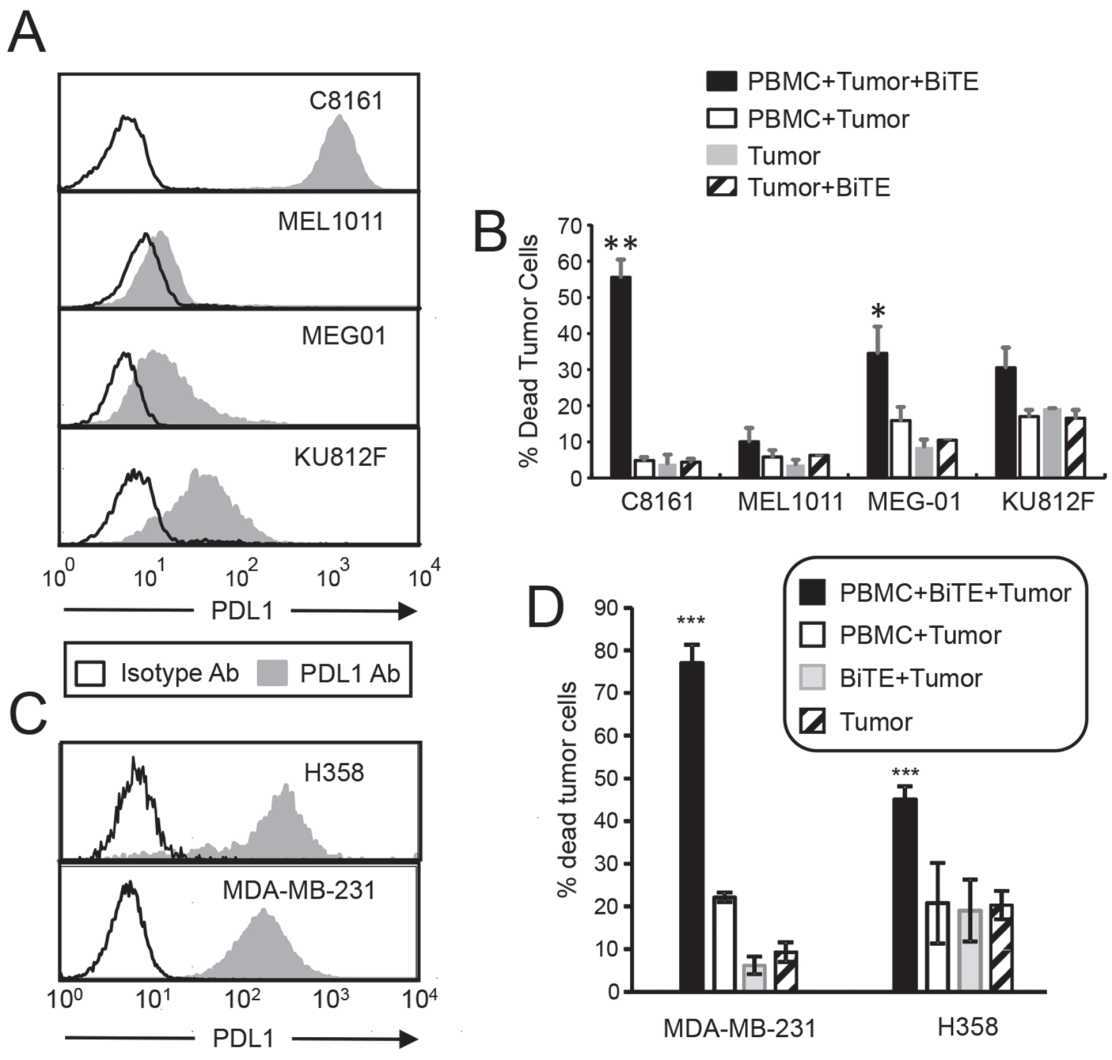
CD3xPDL1 BiTE activates T cells and is cytotoxic for PDL1 **^+^** CML, NSCLC, and breast cancer cells **A**. Tumor cells were incubated for 48 hrs with 200 units human recombinant IFNγ and stained with antibody to PDL1 (29E.2A3 mAb). **B**. Tumor cells were treated with IFNγ as in panel A and then labeled with CellTrace Violet and incubated ± healthy donor PBMC (20:1 ratio PBMC:tumor; 200ng BiTE/ml) ± CD3xPDL1 BiTE and analyzed 72 hrs later for percent dead tumor cells. Values are the average + SE of 5, 3, 4, and 3 independent experiments for C8161, MEL1011, MEG-01, and KU812F cells, respectively. **C**. H358 and MDA-MB-231 tumor cells were stained with fluorescent antibodies to PDL1 (29E.2A3 mAb) or isotype control mAb. **D**. Cytotoxicity assay as in B except H358 and MDA-MB-231 cells were targets. Values for H358 are the average ± SE of 7 independent experiments. Values for MDA-MD-231 are average ± SD of 3 replicates. Values with one or more asterisks are significantly different from other values for the same cell line.

To determine if the BiTE is globally effective against PDL1^+^ tumor cells, two lines of chronic myelogenous leukemia (CML) were tested as target cells. MEG01 and KU812F CML cells do not constitutively express PDL1. However, following treatment with 200 units of recombinant human IFNγ ~21% and ~30% of these cells, respectively, express PDL1, although the magnitude of PDL1 expression is considerably lower than C8161 levels (Figure 4A). PBMC from healthy donors were incubated for 72 hrs with CellTrace Violet-labeled IFNγ-treated MEG01, KU812F, C8161, and MEL1011 cells, with or without BiTE, and the resulting cells were stained with the viability dye 7AAD. The BiTE mediated killing of ~35% and 30% of MEG-01 and KU812F cells, respectively (Figure 4B). Background killing in the absence of BiTE was ~15-18%. To further confirm that the BiTE targets PDL1^+^ tumor cells regardless of the type of tumor, constitutively PDL1^+^ H358 lung adenocarcinoma cells and MDA-MB-231 breast cancer cells (Figure 4C) were also tested and exhibited >45% tumor cell death (Figure 4D). These studies demonstrate that a variety of tumor cell types with a range of PDL1 expression can be killed by BiTE-activated PBMC.

### CD3xPDL1 BiTE facilitates cytotoxicity even at low levels of bite binding to PDL1^+^ tumor cells

**Figure 5:**
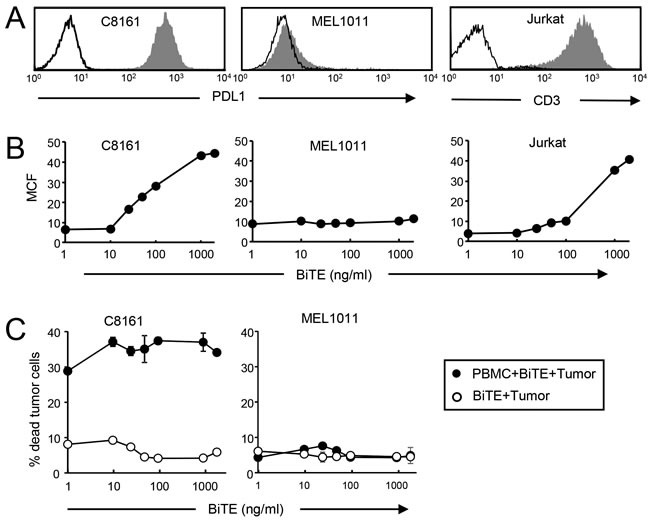
Low levels of bound BiTE are sufficient for maximal killing of PDL1 **^+^** tumor cells **A**. C8161 melanoma, MEL1011 melanoma, and Jurkat cells were labeled with fluorescently-tagged antibodies to PDL1 or CD3 and the corresponding isotype control mAbs. **B**. C8161, MEL1011, and Jurkat cells were treated with titered amounts of CD3xPDL1 BiTE followed by anti-his mAb. **C**. CellTrace Violet-labeled C8161 and MEL1011 cells were incubated with healthy donor PBMC plus titered amounts of BiTE as in fig. 3D. Values are from two independent experiments.

To determine if target cells and effector cells must be saturated with the CD3xPDL1 BiTE for maximum cytotoxicity, we stained PDL1^+^ C8161, PDL1^-^ MEL1011, and CD3^+^ Jurkat cells with increasing amounts of BiTE (Figure 5A, 5B) and then used the same quantity of BiTE in cytotoxicity assays (Figure 5C). If T cells and target cells must be saturated with BiTE, then percent cytotoxicity will decrease if effector cells and target cells bind less than saturating amounts of BiTE. Maximum BiTE binding to melanoma cells and Jurkat cells, respectively, was in the range of 500-2000 ng BiTE/mL and decreased when BiTE concentration was less than 100 ng BiTE/mL. In contrast, PBMC-mediated cytotoxicity of tumor cells was relatively unchanged from 10ng BiTE/mL to 2000 ng BiTE/mL. These results indicate that maximum cytotoxicity does not require high levels of BiTE binding to either target cells or effector cells.

### The BiTE activates CD4^+^ and CD8^+^ cytotoxic T cells and NKT cells

**Figure 6:**
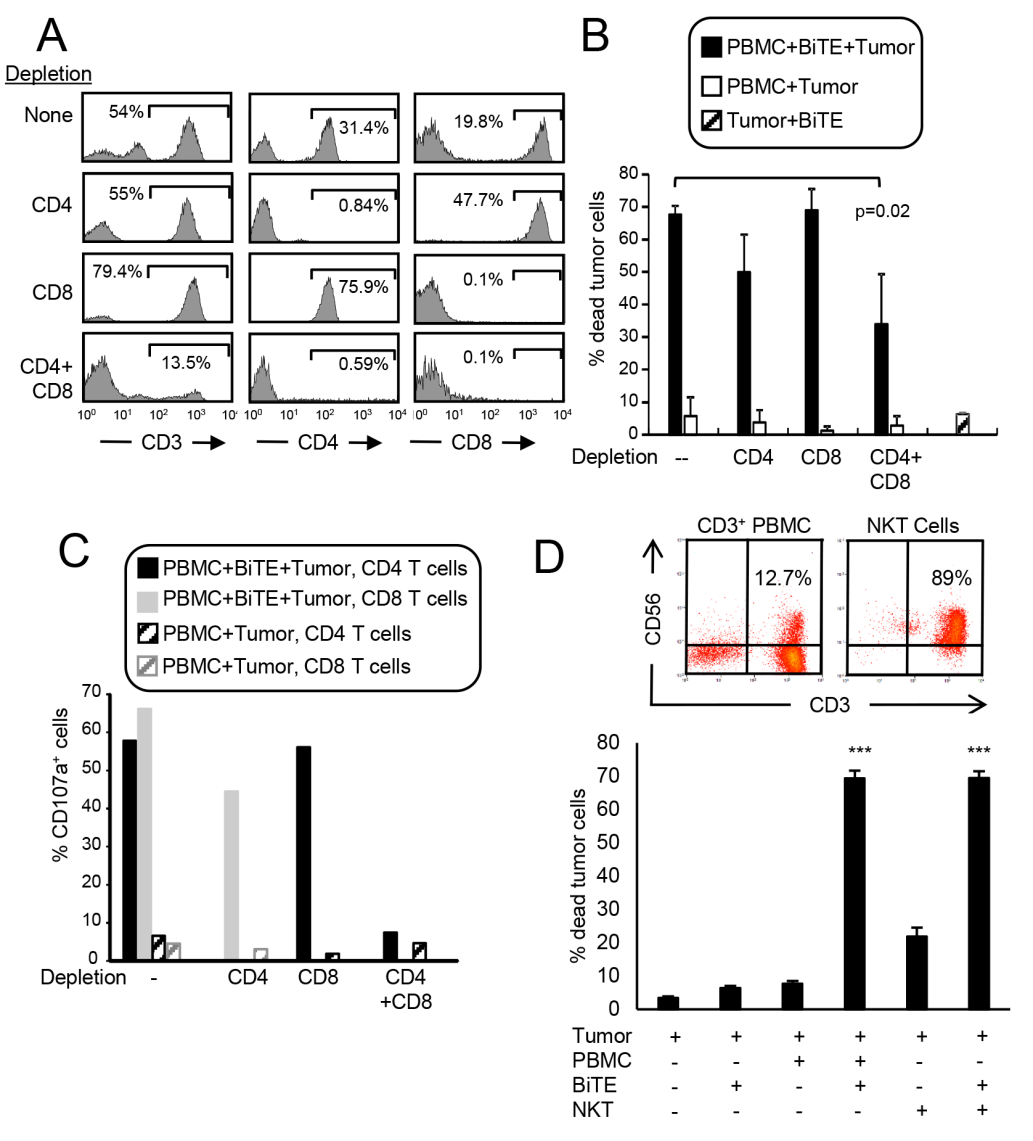
CD3xPDL1 BiTE activates both CD4 **^+^** and CD8**^+^** cytotoxic T cells and NKT cells **A**. PBMC from healthy human donors were either undepleted, or depleted for CD4^+^, CD8^+^, or CD4^+^ plus CD8^+^ T cells. Depleted populations were stained with fluorescently tagged antibodies to CD3, CD4, and CD8, and the CD3^+^ cells gated and analyzed for CD4 and CD8 expression. **B**. Violet-labeled PDL1^+^ H358 tumor cells were incubated with the depleted or not depleted PBMC ± BiTE (200ng/mL), and analyzed for % dead tumor cells. PBMC:tumor cell ratio is 20:1 for undepleted and depleted populations. For the undepleted samples the ratio of CD8 and CD4 T cells to tumor was 5.45:1 and 8.04:1, respectively. In the CD8-depleted and CD4-depleted samples the ratio of CD4:tumor was 16.08:1 and the ratio of CD8:tumor was 11.52:1. Data are the average of three independent experiments. **C**. The cells of panel B were stained with fluorescent antibodies to CD4, CD8, and CD107a and the CD4 and CD8 cells gated and analyzed for CD107a expression. Data are representative of one of two independent experiments. **D**. Violet-labeled ± C8161 tumor cells were incubated with magnetic bead purified CD3^+^ cells (PBMC) or purified CD3^+^CD56^+^ NKT cells ± BiTE (200ng/mL). Data are representative of three independent experiments. Values with * are significantly different from values without *.

Optimal cell-based immunotherapy should activate the maximum number of effector cells. CD8^+^ T cells are traditionally considered the optimal effector cells. However, the BiTE has the capacity to activate any CD3^+^ cell so CD3^+^CD8^+^ and CD3^+^CD4^+^ T cells, as well as CD3^+^ NKT cells may become cytotoxic effector cells. To determine if CD4^+^ and/or CD8^+^ T cells become cytotoxic, healthy donor PBMC were depleted for CD4^+^, CD8^+^, or CD4^+^ plus CD8^+^ cells and subsequently tested for cytotoxic activity in the presence of BiTE and PDL1^+^ C8161 human melanoma cells. Depleted populations contained <1% of the depleted cell population (Figure 6A). To be consistent with earlier experiments, all samples were tested at a 20:1 ratio of PBMC to tumor cells. Ratios of CD8:tumor cells and CD4:tumor cells in the undepleted samples were 5.45:1 and 8.04:1 respectively. In the CD4-depleted and CD8-depleted samples, the ratios of CD8:tumor cells and CD4:tumor cells was 11.52:1 and 16.08:1, respectively. Simultaneous depletion of CD4^+^ plus CD8^+^ T cells reduced killing by 61% (p<0.02), while single depletion of CD4^+^ or CD8^+^ T cells did not significantly reduce cytotoxicity (Figure 6B). Since equal numbers of the depleted populations were tested and depletion of one cell population results in increased numbers of the other population, these results indicate that both CD4^+^ and CD8^+^ T cells are rendered cytotoxic by the BiTE. To further ascertain that both CD4^+^ and CD8^+^ T cells have cytotoxic activity, the cells of panel B were stained with fluorescently-tagged antibodies to CD3, CD4, CD8, and CD107a (Figure 6C). Gated CD3^+^CD4^+^ and CD3^+^CD8^+^ populations contained >45% CD107a^+^ cells indicating they contain granules releasing perforin which is responsible for target cell lysis.

Simultaneous depletion of CD4^+^ and CD8^+^ T cells did not eliminate cytotoxicity, suggesting that other CD3^+^ cells in the PBMC might also be activated by the BiTE. Since NKT cells are CD3^+^, we purified CD3^+^CD56^+^ NKT cells and assessed their ability to kill PDL1^+^ C8161 human melanoma cells in the presence or absence of BiTE (Figure 6D). To be consistent with earlier experiments, all samples were tested at a 20:1 ratio of PBMC to tumor cells. In the presence of BiTE, NKT cells were just as cytotoxic for C8161 cells as total CD3^+^ PBMC. These results confirm that the BiTE renders both CD4^+^ and CD8^+^ T cells and NKT cells cytotoxic for PDL1^+^ target cells.

### The CD3xPDL1 BiTE activates cytotoxic PBMC from cancer patients

**Figure 7:**
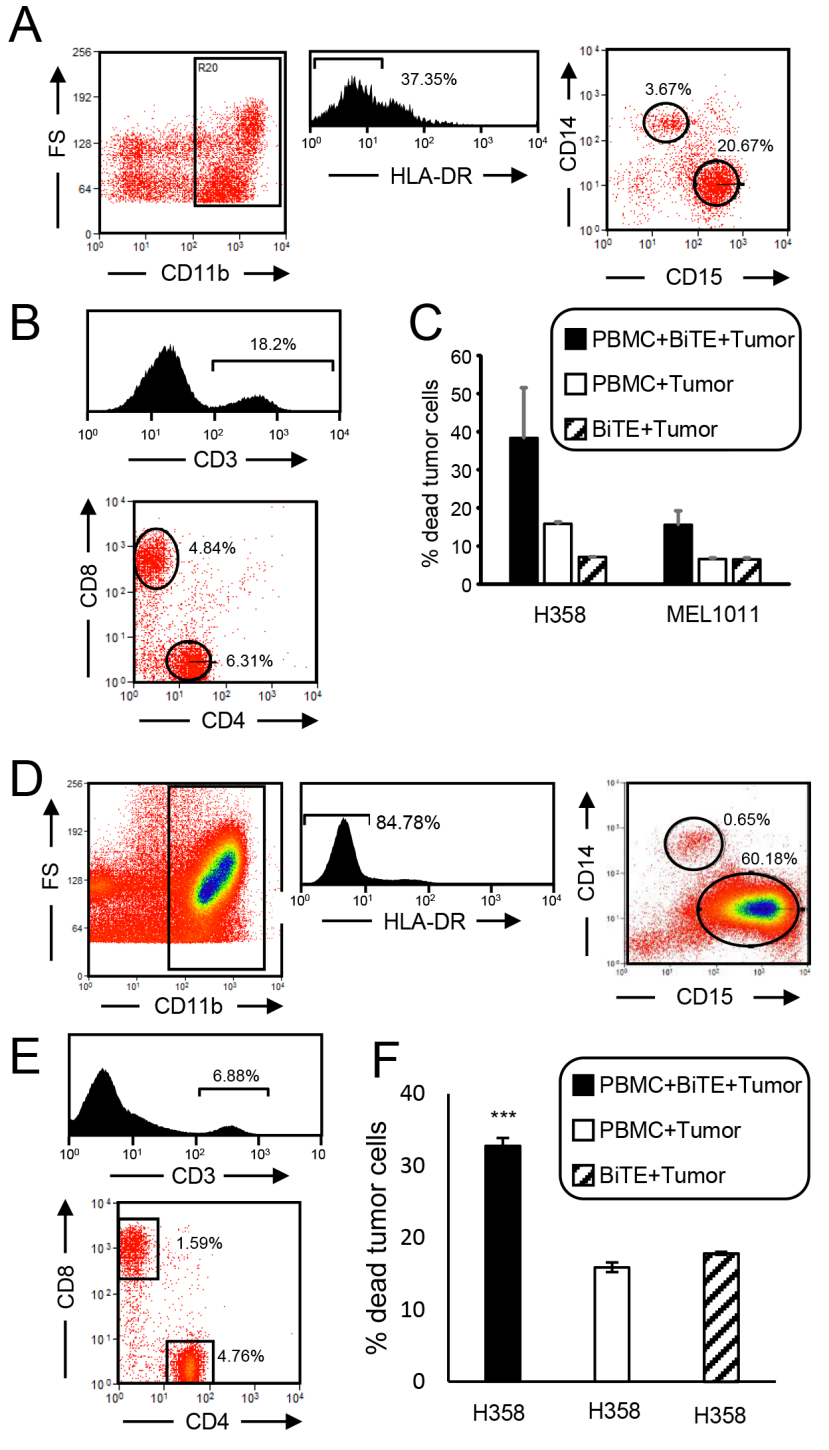
CD3xPDL1 BiTE activates cancer patients’ PBMC that are cytotoxic for PDL1 **^+^** lung cancer cells **A**. PBMC from a SCLC patient were stained for MDSC (CD11b, CD14, CD15, HLA-DR). CD11b^+^HLA-DR^-^ cells were gated and analyzed by flow cytometry for CD14^+^CD15^-^ M-MDSC and CD15^+^CD14^-^ PMN-MDSC. Data are representative of one of three SCLC patients. **B**. SCLC patient's PBMC were labeled with fluorescently-tagged antibodies to CD3, CD4 and CD8, and the gated CD3^+^ cells were analyzed for percent CD3^+^CD4^+^ and CD3^+^CD8^+^ cells. Data are for one of three SCLC patients. **C**. PBMC from the SCLC patients were incubated ± CellTrace violet-labeled PDL1^+^ H358 or PDL1^-^ MEL1011 tumor cells ± BiTE at a 20:1 ratio of PBMC:tumor cells. Cells were stained with 7AAD and the CellTrace violet stained tumor cells were gated and analyzed for % dead cells. The ratio of CD8^+^ plus CD4^+^ T cells to tumor cells is 2.3:1. Data are pooled from two independent experiments with PBMC from two individual patients. **D**. PBMC from a NSCLC patient were stained for MDSC. CD11b^+^HLA-DR^-^ cells were gated and analyzed for CD14^+^CD15^-^ M-MDSC and CD15^+^CD14^-^ PMN-MDSC. **E**. NSCLC patient's PBMC were labeled with fluorescently-tagged antibodies to CD3, CD4 and CD8, and the CD3 gated cells were analyzed for percent CD3^+^CD4^+^ and CD3^+^CD8^+^ cells. **F**. PBMC from the NSCLC patient were incubated ± CellTrace violet-labeled H358 tumor cells ± BiTE at a 20:1 ratio of PBMC:tumor cells. Cells were stained with 7AAD and the CellTrace violet stained tumor cells were gated and analyzed for % dead cells.

Since the goal of these studies is to determine if the CD3xPDL1 BiTE will be efficacious in cancer patients, we have tested PBMC from patients with small cell lung cancer (SCLC) and non-small cell lung cancer (NSCLC). MDSC are frequently present in the PBMC of SCLC and NSCLC patients [27, 28] and could reduce the function of the CD3xPDL1 BiTE. Using the accepted markers for identifying human MDSC [29], PBMC from three SCLC patients were screened by flow cytometry for MDSC. The leukocytes of these patients contained an average of 37.0% ± 11.9 total MDSC, 1.5% ± 1.4 monocytic MDSC (M-MDSC), and 35.5% ± 12.8 granulocytic MDSC (PMN-MDSC). A representative profile of a SCLC patient's PBMC stained for MDSC is shown in Figure 7A. The percent of PBMC that were CD3^+^CD4^+^ and CD3^+^CD8^+^ T cells was also determined (Figure 7B). To determine if the patients’ PBMC could be activated by the BiTE, H358 lung adenocarcinoma cells were labeled with CellTrace Violet and incubated with SCLC patient PBMC with or without BiTE. Approximately 38% of the PDL1^+^ H358 cells vs. ~15% of the PDL1^-^ MEL1011 melanoma cells were killed when incubated with PBMC plus BiTE (Figure 7C). The PBMC to tumor ratio was 20:1 in the cytotoxicity assay; however, since CD8^+^ plus CD4^+^ T cells are only 11.15% of the PBMC, the actual T cell to tumor cell ratio was 2.3:1.

PBMC from a non-small cell lung cancer (NSCLC) patient were also tested. Approximately 60% of this patient's leukocytes were PMN-MDSC, 1.4% were M-MDSC (Figure 7D), 4.76% were CD3^+^CD4^+^, and 1.59% were CD3^+^CD8^+^ (Figure 7E). Similar to the SCLC patient, BiTE treatment of the NSCLC patient's PBMC also resulted in significant cytotoxicity of PDL1^+^ C8161 tumor cells (Figure 7F).

These results demonstrate that the BiTE activates T cells from cancer patients despite the presence of MDSC, and that ratios less than four T cells per tumor cell are needed for killing.

### CD3xPDL1 BiTE significantly extends the survival time of humanized tumor-bearing NSG mice

**Figure 8:**
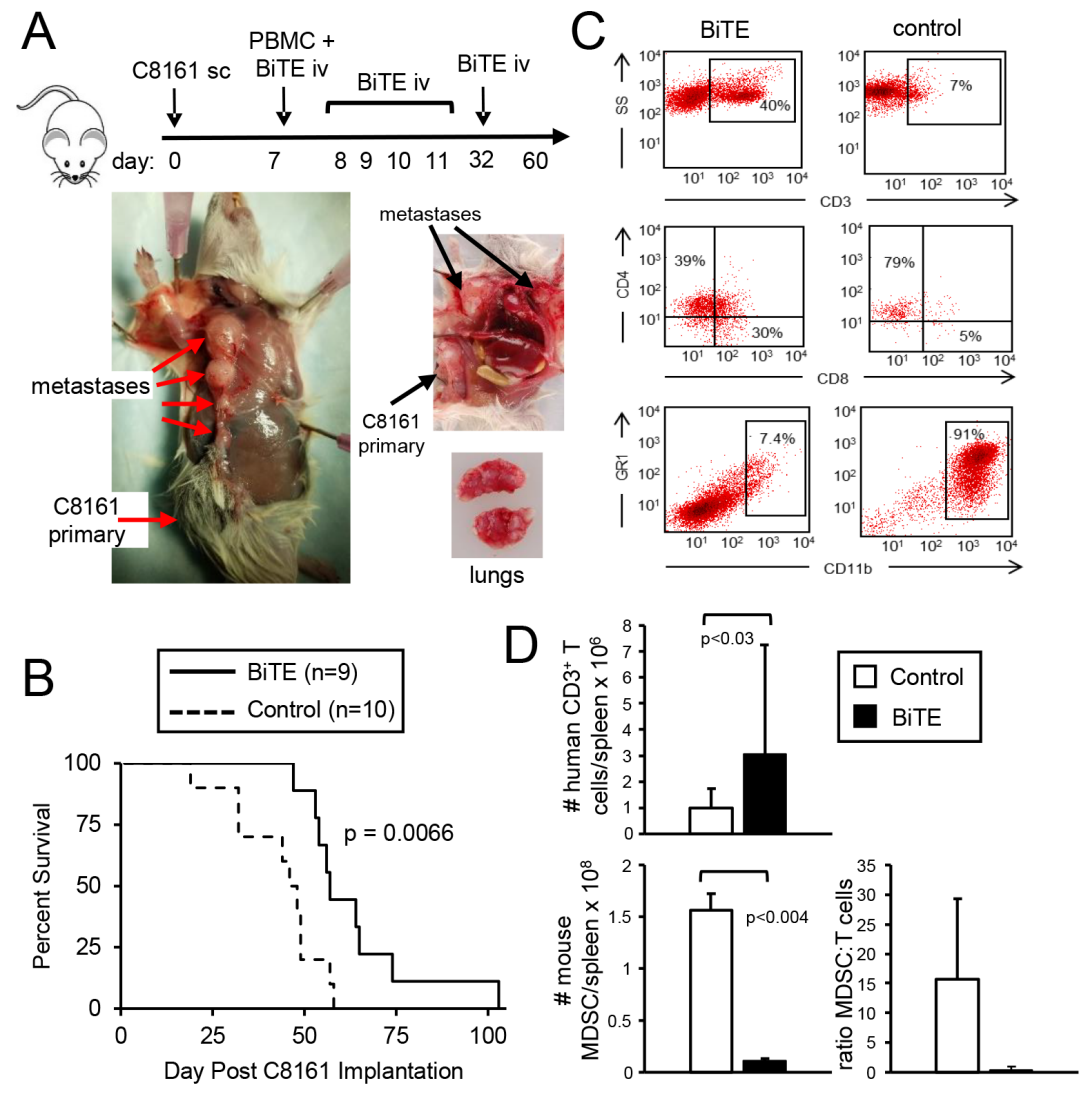
CD3xPDL1 BiTE significantly extends the survival time of humanized NSG mice reconstituted with human PBMC and carrying established metastatic human melanoma C8161 NSG mice were inoculated subcutaneously in the right flank with 1×10^6^ human C8161 melanoma cells on day 0. On day 7 when tumors were palpable, mice were either untreated or administered 1×10^7^ human PBMC iv in the tail vein and 0.2ng (8ng/kg) CD3xPDL1 BiTE iv in the retro-orbital sinus. On days 8, 9, 10, 11, and 32 mice were given additional iv injections of 0.2μg BiTE. **A**. Moribund, euthanized control mouse (tumor + PBMC, no BiTE) showing metastases in the lymph nodes and lungs on day 57. **B.** Kaplan-Meier plot showing survival. Data are pooled from three independent experiments. **C**. Representative flow cytometry profiles of splenocytes of moribund/dead BiTE-treated and untreated mice stained for human T cells (CD3, CD4, and CD8 mAbs), or mouse MDSC (Gr1 and CD11b mAbs). **D**. Total numbers and ratio of human T cells and mouse MDSC in the spleens of moribund/dead BiTE-treated and control mice of panel B. Data are pooled from 2-3 mice per group.

To determine if the CD3xPDL1 BiTE has *in vivo* efficacy, we have used humanized NSG mice [30]. NSG mice are immune deficient and are readily engrafted by human PBMC and human tumor cells, and therefore serve as a model for studying the *in vivo* effects of the human immune system on human tumors. NSG mice were inoculated s.c. in the right rear flank with 1×10^6^ PDL1^+^ human C8161 melanoma cells. When tumors were palpable (day 7), mice were reconstituted with healthy donor human PBMC. Treated mice received five daily injections of BiTE starting on day seven. Control mice did not receive BiTE (Figure 8A). Engrafted mice did not display symptoms of graft-vs.-host disease or symptoms of autoimmunity during the course of the experiments. C8161 is spontaneously metastatic in the reconstituted NSG mice as evidenced by metastatic disease in multiple sites including lymph nodes and lungs. Mean survival time ± SE of BiTE-treated mice was significantly longer than MST of control mice (63.66 ± 5.58 vs. 43.3 ± 3.86 days, respectively; *p* = 0.005) (Figure 8B). A subset of these mice were also followed for growth of primary tumor. There was no difference in progression of primary tumor between the BiTE and control-treated groups (Supplementary Figure 4). The spleens of this latter subset were analyzed at day 54 (42 days after the last BiTE treatment and 47 days after transfer of the PBMC) for their content of CD3^+^, CD4^+^, and CD8^+^ T cells. Representative profiles are shown in Figure 8C, and values are quantified in Figure 8D. The spleens of the control-treated mice were much larger and contained 10 times as many cells as the spleens of BiTE-treated mice (1.6×10^8^ vs. 1.6×10^7^ cells, respectively). Since such spleen enlargement is characteristic of the accumulation of MDSC, splenocytes of the mice were stained for phenotypic markers of both human and mouse MDSC (Figure 8C). Human MDSC were not present (Supplementary Figure 5); however, ~90% of the splenocytes in the control mice were phenotypically mouse MDSC (Gr1^+^CD11b^+^ cells), while the spleens of BiTE-treated mice contained many fewer mouse MDSC. Although the overall number of splenic CD3^+^CD4^+^ and CD3^+^CD8^+^ T cells in control and BiTE-treated mice was similar, the ratio of MDSC to T cells in control mice was significantly greater than the ratio in BiTE-treated mice. These results demonstrate that the CD3xPDL1 BiTE extends the survival time of humanized mice with established, spontaneously metastatic PDL1^+^ tumor cells.

## DISCUSSION

The studies described here demonstrate that the CD3xPDL1 BiTE binds simultaneously to T cell-expressed CD3 and tumor cell-expressed PDL1. In conjunction with PBMC, the CD3xPDL1 BiTE facilitates the specific lysis of PDL1^+^ tumor cells by activating both CD4^+^ and CD8^+^ cytotoxic T cells and NKT cells. *In vivo* studies demonstrated that a short treatment with the CD3xPDL1 BiTE significantly extended the survival time of humanized NSG mice carrying an established metastatic human melanoma. Our rational for generating this particular BiTE was that it would serve the dual purpose of activating anti-tumor immunity while concurrently minimizing PD1-mediated immune suppression.

Binding of the CD3xPDL1 BiTE to CD3^+^ and PDL1^+^ cells is roughly linearly proportional to the amount of CD3 or PDL1 expressed by the target cells. In contrast, PBMC-mediated BiTE killing of target cells does not require high levels of PDL1 since low levels of BiTE binding facilitate similar levels of target cell killing as higher levels of BiTE binding. Although some tumor cells constitutively express PDL1, other tumor cells only express PDL1 after induction by IFNγ. Such induction occurs in the tumor microenvironment when solid tumors are infiltrated by activated IFNγ-secreting T cells [31]. Therefore, the CD3xPDL1 BiTE may be effective in activating antitumor immunity against tumor cells that constitutively express low levels of PDL1 as well as in situations where there is already an active T cell-mediated antitumor immune response and IFNγ is driving the expression of PDL1.

The studies with PBMC from lung cancer patients demonstrated that T cells activated by the CD3xPDL1 BiTE are not inhibited by MDSC, suggesting that patients with high levels of MDSC may be candidates for BiTE therapy. Resistance to MDSC could have occurred because some MDSC express PDL1 and the BiTE mediates lysis of these cells. In addition to MDSC, there are other immune suppressive mechanisms that are active in individuals with cancer and which have the potential to interfere with BiTE function. BiTEs are known to convert existing Tregs to T cytotoxic cells [9]. In the case of the CD3xEGFRvIII BiTE, the conversion of Tregs to cytotoxic effector cells occurred by induction of granzyme and perforin in the Tregs [36]. Therefore, as for other BiTEs, the CD3xPDL1 BiTE may be effective in individuals with high levels of Tregs. Whether BiTE-activated T cells are protected against immune suppressive soluble molecules such as indole-amine 2,3-dioxygenase (IDO) [37], TGFβ [38], or other soluble molecules is not known. In addition to immune suppression by MDSC and Treg-specific mechanisms, tumor-associated macrophages, fibroblasts, mast cells, B cells, and T cells themselves within solid tumors may become PDL1^+^ in response to IFNγ [31]. *In vitro* studies using a combination of a CD3xCD33 BiTE with anti-PDL1 antibodies demonstrated that blockade of the PD1/PDL1 axis augmented BiTE-mediated killing of AML cells, demonstrating that BiTE activation does not by itself overcome PD1-mediated suppression [39]. Since the CD3xPDL1 BiTE targets PDL1^+^ cells, it may also minimize PD1 pathway immune suppression that is likely to escalate as T cell anti-tumor immunity and levels of IFNγ increase in the tumor microenvironment.

However, activated T cells may also express PDL1, so the CD3xPDL1 BiTE may also lyse some of these cells. This activity could limit the effector cell repertoire and thereby counter-act antitumor immunity. However, this potential “off-tumor” effect has not prevented the BiTE from significantly extending survival time in the humanized mouse studies described here.

Human myeloid cell subsets differentiate and accumulate in NSG mice reconstituted with human hematopoietic stem cells [40]; however, the PBMC-reconstituted NSG mice in the current study contained only very low levels of human MDSC. In contrast, the control humanized tumor-bearing NSG mice contained very high levels of mouse MDSC. This finding is not unexpected since the mutations in NSG mice do not affected mouse myeloid cell differentiation [41]. What is surprising, however, is that the BiTE-treated mice have many fewer mouse MDSC and a much lower ratio of mouse MDSC to human T cells. MDSC levels are typically regulated by tumor burden since MDSC are induced by tumor-secreted factors [19]. However, the difference in MDSC levels between control and BiTE-treated mice is not due to differences in the amount of tumor since MDSC levels were determined when both groups were moribund and had similar tumor burdens. The CD3xPDL1 BiTE does not react with mouse PDL1^+^ cells, so the BiTE's effect is not a direct killing of PDL1^+^ mouse MDSC. The CD3xPDL1 BiTE could be down-regulating tumor-induced inflammation and thereby diminishing the accumulation of MDSC since inflammation is a major driving force for MDSC [42]. Whether the relative reduction in MDSC in the BiTE-treated mice contributes to the extended MST is unclear since it is not known if mouse MDSC cross-species barriers and suppress human T cells.

Although the BiTE-treated humanized mice had a significantly extended survival time, they eventually died from metastatic disease. We are unaware of other studies that have tested T cell-based immunotherapies in humanized mice with spontaneously metastatic tumors, so it is not possible to compare the efficacy of the CD3xPDL1 BiTE to other immunotherapies. Extensive work has demonstrated that BiTEs have a very short half-life *in vivo* and that therapeutic efficacy requires continual infusion [43]. The CD3xPDL1 BiTE was administered once a day for five days starting when tumors were palpable (Day 7 after transplant), and a sixth treatment on day 32. Therefore, BiTE was not present during later stages of tumor progression. Given the kinetics of tumor growth and metastasis, it is likely that this scheduling prolonged survival because it delayed or perturbed the metastasis process.

BiTEs have shown efficacy in the clinic and in general are well tolerated. However, clinical responses in some patients are accompanied by significant adverse effects. In three clinical trials, some of the acute lymphoblastic leukemia patients treated with Blincyto (CD3xCD19 BiTE) experienced central nervous system or cytokine release syndrome (CRS) adverse events [44]. Less severe CRS can be treated with the glucocorticoid dexamethasone [44] which is most likely effective because it decreases cytokine production but does not inhibit BiTE-mediated cytotoxic activity [11]. We observed no adverse effects in the NSG-treated mice; however, such effects rarely occur and are difficult to identify in mice. Studies with cancer patients will be necessary to definitively determine the safety of the CD3xPDL1 BiTE.

The studies reported here suggest that the CD3xPDL1 BiTE is a novel reagent that because of its dual ability to activate tumor-reactive T cells and NKT cells and direct them to PDL1^+^ target cells, may be a useful therapeutic. Since not all tumor cells are, or become, PDL1^+^, the CD3xPDL1 BiTE is likely to be most useful in combination with other immunotherapies that have modest immune cell activation activity and do not have the capability of neutralizing PD1-mediated immune suppression.

## MATERIALS AND METHODS

### Construct design and generation of recombinant protein

V_L_, V_H_ regions of the human 12A4 anti-human PDL1 monoclonal antibody [26] and V_H_ and V_L_ regions of the OKT3 anti-human CD3 monoclonal antibody [25] were linearly assembled including 15 base linkers between each segment and a 6 residue his tag at the 3’ end (Supplementary Figure 1A). The OKT3 antibody was originally a mouse mAb but has been humanized [25]. EcoR1 and Nhe1 restriction sites at the 5’ and 3’ ends, respectively, served as sites for cloning the BiTE into the pINFUSE-hIgG1-Fc1 vector (Supplementary Figure 1B). The construct was transformed into DH5α cells and transformed DH5α cells were selected using 25 μg/ml zeocin (Invitrogen). Accuracy of the construct was confirmed by double digestion with NheI-HF and EcoRI-HF, and by sequencing (Genewiz) using pINFUSE preclone sense, PDL1 seq and pINFUSE 6x antisense primers (Supplemental Figures 1C, 1D). The BiTE construct was transfected into CHO cells by Amaxa electroporation [28] and transfectants were selected using zeocin (400 ug/ml; Invitrogen). A high expressing BiTE/CHO clone (H4) was obtained by limiting dilution cloning. Transfectants were expanded in IMDM supplemented with 1% AA, 1% β-mercaptoethanol, 1% Glutamax, 0.1% Gentamicin and 400ug/mL Zeocin. To obtain BiTE for experiments, transfectants were plated at 3×10^7^ cells/30 mL of serum-free HL1 medium (Lonza, Biowhittaker) supplemented with 1% AA, 1% Glutamax, and 0.1% Gentamicin and cultured for 2-4 days at 37°C. Supernatants were then collected, centrifuged to remove cellular debris, and 120ml aliquots were concentrated 20 fold to 6 mL using Ultra-15 10kD Centrifugal Filter Units (Millipore/Amicon) to eliminate molecules >10KD. BiTE was identified by western blotting with anti-6xhis mAb (AD1.1.10, Invitrogen), and quantified by comparing band density of slot-blotted BiTE to a his-tagged standard protein (CD80-Fc, R&D) using ImageJ software. BiTE was stored at 4°C until used. Concentrated supernatant from BiTE-transfected CHO cells and similarly concentrated supernatant from non-BiTE transfected CHO cells were electrophoresced on denaturing 12% SDS-PAGE gels, and the resulting gels were stained with Coomassie blue dye to assess purity (Supplementary Figure 1E). BiTE supernatants contained a prominent band at 55kD and a less prominent band of unknown identification at ~70kD. The ~70kD band, but not the ~55kD band, was also present in the non-BiTE supernatants.

### Cells, antibodies, control recombinant proteins, healthy donor and patient PBMC

CHO, CML lines MEG01 [45] and KU812F [46] cells were obtained from the ATCC. CHO cells were cultured in IMDM medium supplemented with 10% FCS, 1% Glutamax, 1% gentamycin, 1% penicillin, and 1% streptomycin. KU812F and MEG01 cells were cultured in RPMI 1640 supplemented with 10% FCS, 1% Glutamax, 1% gentamycin, 1% penicillin, and 1% streptomycin or serum-free HL1 medium supplemented as per IMDM medium. Jurkat cells were cultured in IMDM supplemented with 10%FCS, 1% penicillin-streptomycin, 1% Glutamax, 0.1% gentamycin, and 5×10^-5^M β-mercaptoethanol. Characterization and culturing of human lung adenocarcinoma H358, and melanomas C8161 [47] and 1011 were previously described [28, 48, 49].

Antibodies to human PDL1 (CD274; clone 29E.2A3; BV421 and PE-Cy7), PD1 (clone EH12.2H7, PE-Cy7), CD3 (clone OKT3, FITC, PE, APC), CD3 (clone UCHT1, BV421), CD4 (clone OKT4, PB, BV510, APC, APC-Cy7, PE-Cy7), CD8 (clone SK1, APC), CD8a (clone HIT8a, PE), CD69 (clone FN50, AlexaF488), HLA-DR (clone L243, BV421), CD14 (clone HCD14, FITC), CD15 (clone HI98, PE), CD11b (clone ICRF44, APC), CD56 (clone HCD56, FITC), LEAF purified anti-human CD3 (clone OKT3), LEAF-purified anti-human PDL1 (clone 29E.2A3) were from BioLegend. Antibodies to human PD1 (clone MIH4, PE), PDL1 (clone MIH1, PE), CD25 (clone M-A251, APC-Cy7), CD80 (clone L307.4, PE), CD80 (clone L307.4, FITC), and CD25 (clone M-A251, PE-Cy7, APC-Cy7) were from BD Pharmingen. Antibodies to human PDL1 (clone MIH1, PE-Cy7), CD4 (clone OKT4, FITC, eFluor605), CD8 (clone OKT8, FITC), and CD8a (clone IT8a, PE) were from eBioscience. Control recombinant protein TROY-Fc was from R&D. Antibodies to mouse CD11b (clone M1/70, APC) and mouse Gr1 (clone Rb6.8C5, PacB) were from BioLegend.

Human PBMC from healthy donors were prepared on ficoll gradients and stored in liquid nitrogen until used as previously described [28]. PBMC used for NKT purification were obtained from AllCells, LLC (Alameda, CA) and were handled according to the manufacturer's direction. Fresh samples of blood from SCLC and NSCLC patients were bled into heparinized tubes and treated with Gey's solution to remove red blood cells. Patient PBMC were not processed on ficoll gradients. The resulting PBMC were used fresh.

### Flow cytometry BiTE binding assays

Target cells were resuspended in HL1 medium and loaded into wells of a 96 well flat bottomed plate (2×10^5^ cells/well). Plates were centrifuged (600g x 3-5 minutes) and the supernatants removed by flicking. Twenty-five to 50 μl of medium (usually HL1) containing BiTE was added to each well and the plates incubated on ice for a minimum of 30 minutes. Cells were then washed twice by addition of 200 μl medium/well, followed by centrifugation and flicking. Thirty-five μl of anti-his antibody (ThermoScientific) was then added to each well and the plates incubated on ice for a minimum of 30 minutes. The cells were then washed twice and resuspended in 100μl medium. If viability was tested, then 15 μl of 7AAD per 400 μl of sample was added. Labeled cells were analyzed using a Beckman Coulter Cyan ADP flow cytometer and Summit software.

### Cytotoxicity assay

Target cells were labeled using a CellTrace Violet kit according to the manufacturer's directions (Molecular Probes). Labeled cells were adjusted to 5 × 10^4^ cells/ml (RPMI) and 100 μl (5,000 cells) were placed in each well of a 96-well round-bottom plate along with 100μl of PBMC in RPMI medium at the indicated ratios. Fifty μl of HL-1 medium containing BiTE or antibodies to OKT3 or PDL1 at the desired concentration were added to each well, and the plates incubated at 37°C, 5% CO_2_. Following a 48 hr incubation, the contents of each well were transferred to 96 well flat-bottomed plates for further analysis. If adherent target cells were used, the wells were trypsinized (50 μl trypsin-EDTA/well; trypsin neutralized with 150 μl HL-1, 5% serum), the cells washed, and added to the wells of the 96 well flat bottom plate. Resulting cells were stained with the indicated antibodies and 7AAD as previously described, and analyzed by flow cytometry on a Cyan ADP flow cytometer with Summit software. % dead tumor cells = [dead tumor cells (violet^+^7AAD^+^)/total tumor cells (violet^+^)] x 100%.

### BiTE-induced T cell activation and proliferation

PBMC were resuspended to 1×10^6^ cells/ml and Violet Tracker-labeled tumor cells to 5×10^4^ cells/ml. PBMC (1×10^5^/100 μl) ± tumor cells (5000/100μl) were incubated ± BiTE or irrelevant recombinant protein (TROY-Fc) in triplicate in a total volume of 250 μl in 96-well flat bottom plates. Following a 24 or 48 hr incubation, cells and supernatants were harvested. IFNγ in supernatants was measured using an ELISA kit according to the manufacturer's directions (R&D). Cells were stained with fluorescent antibodies to CD4, CD8, CD69, and CD25. Violet-labeled tumor cells were gated out, and gated CD4^+^ and CD8^+^ T cells were assessed for expression of activation markers. Violet Tracker-labeled CD3^+^ PBMC were resuspended to 1×10^6^ cells/ml and tumor cells to 5×10^4^ cells/ml. PBMC (1×10^5^/100 μl) ± tumor cells (5000/100μl) were incubated ± BiTE in a total volume of 250 μl in 96-well flat bottom plates. Following a 48 hr incubation, cells were harvested and analyzed for proliferation by flow cytometry on a Cyan ADP flow cytometer with Summit software.

### PBMC depletions and cell isolations

CD4 and CD8 depletions: PBMC (10^7^/250μl) were incubated for 10 minutes at room temperature with 5μg of biotinylated anti-CD4 antibody (clone RPA-T8, BioLegend), or 5μg of biotinylated anti-CD8 antibody (clone SK3, BioLegend), washed twice with excess PBS-2% FCS, resuspended in 250 μl PBS/2% FCS and then depleted using Rapidsphere streptavidin magnetic beads (31.25μl beads/ 250μl of labeled cells) according to the manufacturer's directions (StemCell, Inc). NKT purification: For NKT experiments NKT cells were positively purified and CD3 cells were negatively purified using NKT purification kits according to the manufacturer's directions (Miltenyi Biotech). For other experiments: CD3^+^ PBMC were negatively purified using CD3 purification kits according to the manufacturer's directions (StemCell, Inc). Depletions and purified populations were ascertained by flow cytometry.

### IFNγ induction

Cells were incubated for 48 hrs in their standard medium supplemented with 200 units/ml recombinant human IFNγ (R&D).

### *In vivo* NSG tumor studies

NSG mice were bred and maintained in the UMBC animal facility. All animal procedures were conducted under an approved animal protocol from the UMBC Institutional Animal Care and Use Committee (IACUC). Six to eight week old NSG mice were inoculated on day 0 with 1×10^6^ C8161 melanoma cells. On day seven, when tumors were palpable, mice were adoptively transferred via the tail vein with 1×10^7^ PBMC from healthy human donors. Also on day seven, mice administered via the retro-orbital sinus 0.2ng/mouse (8 ng/kg) CD3xPDL1 BiTE. Mice received daily BiTE treatments of 0.2ng/mouse on days 8, 9, 10, 11, and 32. Mice were followed for survival and sacrificed when they became moribund, as required by the UMBC IACUC. Percent total CD3^+^ cells and % MDSC in the spleen = (% CD3^+^CD4^+^ plus CD3^+^CD8^+^ splenocytes as assessed by flow cytometry) x (total number of splenocytes) and %Gr1^+^CD11b^+^ splenocytes as assessed by flow cytometry) x (total number of splenocytes), respectively. Ratio of MDSC:T cells = total number of Gr1^+^CD11b^+^ splenocytes/total number of CD3^+^ splenocytes.

### Surface plasmon resonance (Biacore)

Biacore analysis was performed in the Biosensor Core, Dept. of Physiology, University of Maryland, Baltimore. BiTE binding to PDL1-Fc (ThermoFisher) was determined using a GE Healthcare 3000 instrument and multi-cycle kinetics. The BiTE was immobilized to a CM5 chip with anti-His antibodies and then soluble PDL1-Fc (0-5 nM) was passed over the chip. Chi square = 0.264. BiTE binding to CD3 was determined using a GE Healthcare T200 instrument and single-cycle kinetics. CD3εδ heterodimer (Acro Biosystems) was immobilized to a CM5 chip using the amine coupling method in which the carboxylmethyl-dextran surface of the chip was activated with a 35μl injection of a mixture of 0.1 M NHS and 0.1 M EDC in water. BiTE (0-10nM) was injected without regeneration between each injection cycle. Chi square = 0.421. A blank flow cell was used as a reference. The KD was calculated by using a 1:1 Langmuir kinetic model. Unless otherwise specified, all reagents were from GE Healthcare (Piscataway, NJ, USA).

### Statistical analyses

Statistical analysis of tumor growth rates was conducted using the compare Growth Curves function of the Statmod software package (http://bioinf.wehi.edu.au/software/compareCurves/). Statistical analysis of Kaplan-Meier graphs was conducted using the Log Rank Test function of the Statmod software package (http://bioinf.wehi.edu.au/software/russell/logrank/). Student's t test was utilized to determine statistical significance between two sets of data using Microsoft Excel Version 2010. p values <0.05 were considered statistically significant. One-way ANOVA with Tukey's post-hoc test was performed using GraphPad Prism version 7 for Windows, GraphPad Software, La Jolla California USA,www.graphpad.com. Error bars represent standard deviation unless noted otherwise. Asterisks in figures indicate that the experimental value is statistically significantly different from the associated controls at * = *p* < 0.05; ** = *p* < 0.01; *** = *p* < 0.001.

## SUPPLEMENTARY MATERIALS FIGURES



## Abbreviations

BiTE: bispecific T cell engager
CML: chronic myelogenous leukemia
i.v.: intravenous
mAb: monoclonal antibody
MDSC: myeloid-derived suppressor cells
MST: mean survival time
NSCLC: non-small cell lung cancer
PD1: programmed death 1
PDL1: programmed death ligand 1
SCLC: small cell lung cancer
s.c.: subcutaneous
sPDL1: soluble PDL1
Tregs: T regulatory cells
