# Supplementary material for: CD3xPDL1 bi-specific T cell engager (BiTE) simultaneously activates T cells and NKT cells, kills PDL1+ tumor cells, and extends the survival of tumor-bearing humanized mice
Source: Oncotarget. 2017 Aug 3;8(35):57964–80. doi: 10.18632/oncotarget.19865 (PMC5601626; doi:10.18632/oncotarget.19865)
Supplement: Supplementary file 1 [file oncotarget-08-57964-s001.pdf]

CD3xPDL1 bi-specific T cell engager (BiTE) simultaneously activates T cells and NKT cells, kills PDL1^{+} tumor cells, and extends the survival of tumor-bearing humanized mice

SUPPLEMENTARY MATERIALS

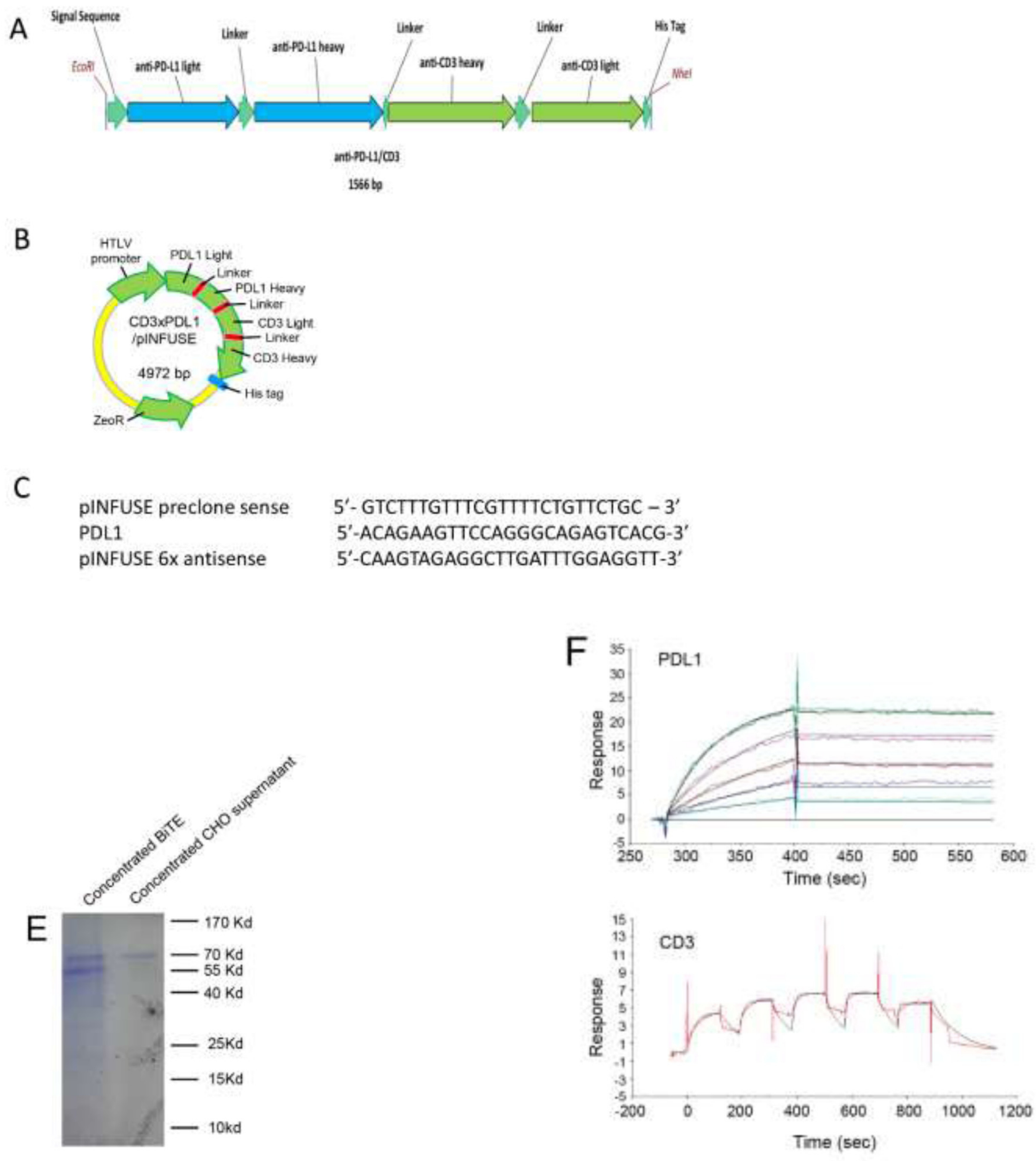

Supplementary Figure 1: CD3xPDL1 BiTE construct and protein. A. Linear assembly of the anti-PDL1 and anti-CD3 V_L and V_H regions including linkers and 5' and 3' restriction sites. **B**. CD3xPDL1 BiTE sequence inserted into the pINFUSE vector. **C**. Primers used for sequencing the CD3xPDL1 BiTE. **D**. CD3xPDL1 BiTE sequence. **E**. Concentrated supernatant from BiTE-transfected CHO cells contains a specific ~55Kd protein. Concentrated supernatants from BiTE-transfected and non-transfected CHO cells were electrophoresced on 12% SDS-PAGE gels and stained with Coomassie blue. F. Biacore analysis of BiTE binding to PDL1 and CD3. PDL1, KD=1.28x10^{-11} (Chi^2=0.264; multiple cycle kinetics and affinity); CD3, KD=2.46x10^{-10} (Chi^2=0.421; single cycle kinetics and affinity).

```
ATG GGA TGG AGC TGT ATC ATC CTC TTC TTG GTA GCA ACA GCT ACA GGT GTC CAC TCC GAA   60
 M   G   W   S   C   I   I   L   F   L   V   A   T   A   T   G   V   H   S   E
ATT GTG TTG ACA CAG TCT CCA GCC ACC CTG TCT TTG TCT CCA GGG GAA AGA GCC ACC CTC   120
 I   V   L   T   Q   S   P   A   T   L   S   L   S   P   G   E   R   A   T   L
TCC TGC AGG GCC AGT CAG AGT GTT AGC AGC TAC TTA GCC TGG TAC CAA CAG AAA CCT GGC   180
 S   C   R   A   S   Q   S   V   S   S   Y   L   A   W   Y   Q   Q   K   P   G
CAG GCT CCC AGG CTC CTC ATC TAT GAT GCA TCC AAC AGG GCC ACT GGC ATC CCA GCC AGG   240
 Q   A   P   R   L   L   I   Y   D   A   S   N   R   A   T   G   I   P   A   R
TTC AGT GGC AGT GGG TCT GGG ACA GAC TTC ACT CTC ACC ATC AGC AGC CTA GAG CCT GAA   300
 F   S   G   S   G   S   G   T   D   F   T   L   T   I   S   S   L   E   P   E
GAT TTT GCA GTT TAT TAC TGT CAG CAG CGT AGC AAC TGG CCG ACG TTC GGC CAA GGG ACC   360
 D   F   A   V   Y   Y   C   Q   Q   R   S   N   W   P   T   F   G   Q   G   T
AAG GTG GAA ATC AAA GGT GGA GGT GGA TCT GGG GGA GGT GGA TCT GGG GGT GGG GGA TCG   420
 K   V   E   I   K   G   G   G   G   S   G   G   G   G   S   G   G   G   G   S
CAG GTC CAA CTG GTG CAG TCT GGG GCT GAG GTC AAG AAG CCT GGG TCG TCG GTG AAG GTC   480
 Q   V   Q   L   V   Q   S   G   A   E   V   K   K   P   G   S   S   V   K   V
TCC TGC AAG ACT TCT GGA GAC ACC TTC AGC ACC TAT GCT ATC AGC TGG GTG CGA CAG GCC   540
 S   C   K   T   S   G   D   T   F   S   T   Y   A   I   S   W   V   R   Q   A
CCT GGA CAA GGG CTT GAG TGG ATG GGA GGG ATC ATC CCT ATA TTT GGT AAA GCA CAC TAC   600
 P   G   Q   G   L   E   W   M   G   G   I   I   P   I   F   G   K   A   H   Y
GCA CAG AAG TTC CAG GGC AGA GTC ACG ATT ACC GCG GAC GAA TCC ACG AGC ACA GCC TAC   660
 A   Q   K   F   Q   G   R   V   T   I   T   A   D   E   S   T   S   T   A   Y
ATG GAG CTG AGC AGC CTG AGA TCT GAG GAC ACG GCC GTG TAT TTT TGT GCG AGA AAG TTT   720
 M   E   L   S   S   L   R   S   E   D   T   A   V   Y   F   C   A   R   K   F
CAC TTT GTT TCG GGG AGC CCC TTC GGT ATG GAC GTC TGG GGC CAA GGG ACC ACG GTC ACC   780
 H   F   V   S   G   S   P   F   G   M   D   V   W   G   Q   G   T   T   V   T
GTC TCC TCA GGA GGT GGT GGA TCC GAT ATC AAA CTG CAG CAG TCA GGG GCT GAA CTG GCA   840
 V   S   S   G   G   G   G   S   D   I   K   L   Q   Q   S   G   A   E   L   A
AGA CCT GGG GCC TCA GTG AAG ATG TCC TGC AAG ACT TCT GGC TAC ACC TTT ACT AGG TAC   900
 R   P   G   A   S   V   K   M   S   C   K   T   S   G   Y   T   F   T   R   Y
ACG ATG CAC TGG GTA AAA CAG AGG CCT GGA CAG GGT CTG GAA TGG ATT GGA TAC ATT AAT   960
 T   M   H   W   V   K   Q   R   P   G   Q   G   L   E   W   I   G   Y   I   N
CCT AGC CGT GGT TAT ACT AAT TAC AAT CAG AAG TTC AAG GAC AAG GCC ACA TTG ACT ACA   1020
 P   S   R   G   Y   T   N   Y   N   Q   K   F   K   D   K   A   T   L   T   T
GAC AAA TCC TCC AGC ACA GCC TAC ATG CAA CTG AGC AGC CTG ACA TCT GAG GAC TCT GCA   1080
 D   K   S   S   S   T   A   Y   M   Q   L   S   S   L   T   S   E   D   S   A
GTC TAT TAC TGT GCA AGA TAT TAT GAT GAT CAT TAC TGC CTT GAC TAC TGG GGC CAA GGC   1140
 V   Y   Y   C   A   R   Y   Y   D   D   H   Y   C   L   D   Y   W   G   Q   G
ACC ACT CTC ACA GTC TCC TCA GTC GAA GGT GGA AGT GGA GGT TCT GGT GGA AGT GGA GGT   1200
 T   T   L   T   V   S   S   V   E   G   G   S   G   G   S   G   G   S   G   G
TCA GGT GGA GTC GAC GAC ATT CAG CTG ACC CAG TCT CCA GCA ATC ATG TCT GCA TCT CCA   1260
 S   G   G   V   D   D   I   Q   L   T   Q   S   P   A   I   M   S   A   S   P
GGG GAG AAG GTC ACC ATG ACC TGC AGA GCC AGT TCA AGT GTA AGT TAC ATG AAC TGG TAC   1320
 G   E   K   V   T   M   T   C   R   A   S   S   S   V   S   Y   M   N   W   Y
CAG CAG AAG TCA GGC ACC TCC CCC AAA AGA TGG ATT TAT GAC ACA TCC AAA GTG GCT TCT   1380
 Q   Q   K   S   G   T   S   P   K   R   W   I   Y   D   T   S   K   V   A   S
GGA GTC CCT TAT CGC TTC AGT GGC AGT GGG TCT GGG ACC TCA TAC TCT CTC ACA ATC AGC   1440
 G   V   P   Y   R   F   S   G   S   G   S   G   T   S   Y   S   L   T   I   S
AGC ATG GAG GCT GAA GAT GCT GCC ACT TAT TAC TGC CAA CAG TGG AGT AGT AAC CCG CTC   1500
 S   M   E   A   E   D   A   A   T   Y   Y   C   Q   Q   W   S   S   N   P   L
ACG TTC GGT GCT GGG ACC AAG CTG GAG CTG AAA CAT CAT CAC CAT CAT CAT TAG           1550
 T   F   G   A   G   T   K   L   E   L   K   H   H   H   H   H   H   *
```

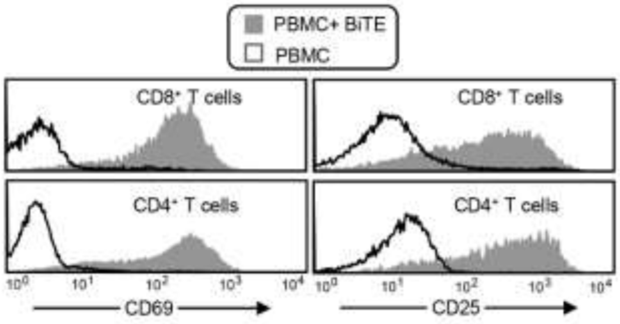

Supplementary Figure 2: The CD3xPDL1 BiTE induces CD4$^+$ and CD8$^+$ T cells to express the activation markers CD69 and CD25. PMBC from a healthy human donor were incubated with the CD3xPDL1 BiTE in the presence or absence of PDL1$^+$ human C8161 tumor cells. Following a 48 hr incubation, the cells were labeled for CD3, CD4, CD8, and CD69 or CD25 and the gated CD3$^+$CD4$^+$ and CD3$^+$CD8$^+$ cells assessed by flow cytometry for CD25 and CD69 expression. Data for CD25 and CD69 are representative of five and two independent experiments, respectively.

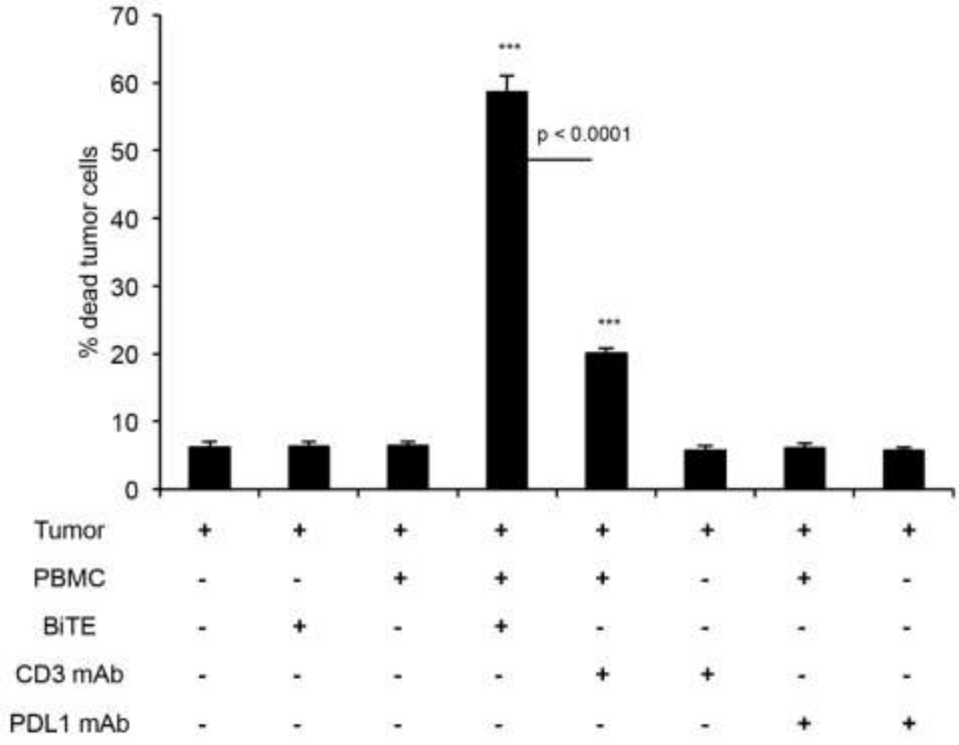

Supplementary Figure 3: The CD3xPDL1 BiTE activates PBMC that are cytotoxic for PDL1$^+$ tumor cells better than a CD3 mAb or PDL1 mAb alone. PDL1$^+$ C8161 melanoma cells were stained with CellTrace Violet and incubated at a 1:20 ratio with healthy donor PBMC ± BiTE, CD3 mAb or PDL1 mAb. Following 48 hrs of incubation, cells were stained with the viability dye 7AAD to identify dead cells. % dead tumor cells = [dead tumor cells (violet$^+$7AAD$^+$)/total tumor cells (violet$^+$)] x 100%. Values are the average of two independent experiments. Error bars indicate standard errors. One-way ANOVA followed by Dunnett's multiple comparisons test or Tukey's multiple comparisons test was performed. Values with asterisks are significantly different from all other values.

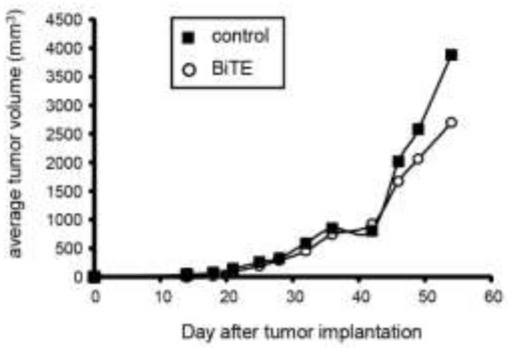

Supplementary Figure 4: CD3xPDL1 BiTE does not impact the growth of primary C8161 melanoma in humanized NSG mice. NSG mice were inoculated s.c in the flank with 1x10^6 C8161 melanoma cells on day 0. Mice were given 1x10^7 healthy donor PBMC i.v. (tail vein) on day 7 when tumors were palpable, and either untreated or treated with 0.2 μg/mouse/injection for 5 consecutive days starting on day 7. Tumors were measured using a calipers.

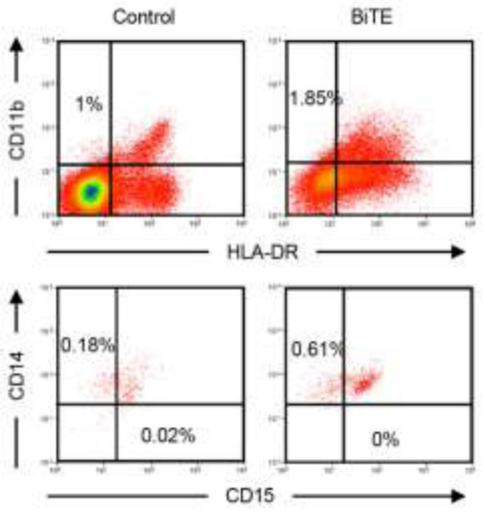

Supplementary Figure 5: Human MDSC are present in very low levels in PBMC-humanized NSG mice and their levels do not differ between control and BiTE-treated mice. Spleens of moribund/sacrificed humanized NSG mice from figure 8 were analyzed by flow cytometry for human MDSC. Profiles show gated $CD11b^{+}$ $HLA^{-}DR^{-}$ cells analyzed for CD14 and CD15. M-MDSC are $CD11b^{+}HLA^{-}DR^{-}$ $CD14^{+}$; PMN-MDSC are $CD11b^{+}$HLA-DR- $CD15^{+}$. Data are representative of two mice/group.
